# Relationship between parathyroid mass and parathyroid hormone level in hemodialysis patients with secondary hyperparathyroidism

**DOI:** 10.1186/s12882-015-0077-6

**Published:** 2015-06-10

**Authors:** Li Fang, Bing Tang, Dawei Hou, Meijuan Meng, Mingxia Xiong, Junwei Yang

**Affiliations:** Center for Kidney Disease, 2nd Affiliated Hospital, Nanjing Medical University, 262 Zhongshan North Road, Nanjing, Jiangsu Province China; Department of General Surgery, 2nd Affiliated Hospital, Nanjing Medical University, 262 Zhongshan North Road, Nanjing, Jiangsu Province China

**Keywords:** Secondary hyperparathyroidism, Total parathyroidectomy, Parathyroid gland mass, Parathyroid hormone

## Abstract

**Background:**

To evaluate the influence of parathyroid mass on the regulation of parathyroid hormone (PTH) secretion, we investigated the relationship between the resected parathyroid gland in total parathyroidectomy and the parathyroid hormone level in hemodialysis patients with secondary hyperparathyroidism.

**Methods:**

From January 2009 to July 2014, 223 patients undergoing total parathyroidectomy were included. The size and the weight of parathyroid gland were measured during the operation.

**Results:**

874 parathyroid glands were removed. A positive correlation was identified between the size and the weight of resected parathyroid glands. We found that both the preoperative PTH and the reduction of PTH were significantly correlated with the size and the weight of parathyroid glands in a positive manner. However, in the subgroup of patients with PTH < 1000 pg/ml, no significant correlation was found.

**Conclusions:**

Larger parathyroid gland secretes more PTH and high level of serum PTH usually indicated that surgical removal might be required. However, since PTH levels could be influenced by the pharmaceutical drug, the large size of parathyroid gland might be used as a much more appropriate guide that indicates the requirement of surgery treatment even when the parathyroid hormone was less than 1000 pg/ml.

## Background

Secondary hyperparathyroidism characterized by increased secretion of PTH, is one of the major serious complications of patients with chronic renal failure on long-term hemodialysis [1]. In the course of secondary hyperparathyroidism, parathyroid growth progresses gradually from diffuse hyperplasia to nodular hyperplasia, and eventually to formation of adenomas at advanced stages [2–4]. Several clinical studies have documented that although PTH levels can be suppressed by continuous treatment with phosphate binders, vitamin D analogs or calcimimetics [5–7], the progressively reducing expression of calcium-sensing receptors (CaSR) and vitamin D receptors (VDR) would result in resistance to treatment [8, 9], and, finally, requiring parathyroidectomy (PTX) [10, 11]. Parathyroidectomy was reported to become gradually necessary [12].

In the preoperative assessment of parathyroidectomy, ultrasonography, scintigraphy, and positron emission tomography scans are used to estimate the glandular size and help plan the operative approach. However, imaging studies are usually heavily operator-dependent, which could even explain the wide variability in the reported sensitivity (ranging from 30–80 %) for the detection of abnormal parathyroid glands [13]. Thus, the surgeons could not be entirely dependent on the imaging techniques. Since the surgical exploration for parathyroid glands and the surgical experience were crucial, successful parathyroidectomy requires an understanding of both the anatomy and the embryology of the parathyroid glands. The surgeons need to learn more about the relationship between the parathyroid glands and the parathyroid hormone level. McCarron et al. have reported that PTH induced by ethylene diamine tetraacetic acid (EDTA) response was related to their total gland size measured at the time of their PTX [14]. However, some studies suggested that the altered quality of the parathyroid mass and not only the increased parathyroid mass might be responsible for non-controllable hyperparathyroidism in uremia [9]. The relationship between parathyroid gland mass and the parathyroid hormone levels remains uncertain. In this study, to evaluate the influence of parathyroid mass on the regulation of parathyroid hormone secretion, we investigated the relationship between the parathyroid gland resected in total parathyroidectomy and the parathyroid hormone levels in hemodialysis patients with secondary hyperparathyroidism.

## Patients and methods

### Selection of patients

We performed a retrospective analysis of a prospectively collected cohort of consecutive adult patients who underwent parathyroidectomy (PTX) treatment between January 2009 and July 2014 in our institution. From January 2009 to July 2014, 223 patients undergoing maintenance hemodialysis therapy with biochemical or clinical evidence, or both, of secondary hyperparathyroidism were referred to the second affiliated hospital of Nanjing medical university for parathyroidectomy. Inclusion criteria were as follows: 1) patients receiving dialysis for more than 6 months; 2) serum intact PTH level >500 pg/mL on two or more occasions; and/or 3) patients with parathyroid nodular or diffuse hyperplasia identified by ultrasound imaging or radioisotope scan; 4) patients with symptomatic secondary hyperparathyroidism, such as bone and joint pain, pathologic fractures, severe pruritus, restless legs syndrome and so on. In all patients, 4 h hemodialysis was performed three times a week with bicarbonate dialysate containing a 3.0 mEq/l calcium concentration. Exclusion criteria included a history of parathyroidectomy, an unstable medical condition during the previous 30 days and the cases with ectopic or residual parathyroid glands.

### Study design

The study protocol was approved by the Ethics Committee of Nanjing Medical University. All patients gave their consent to undergo PTX and participated in this study. The written informed consent was obtained and documented in the legal medical record. In the preoperative preparation, all cases had routine preoperative tests including complete blood counts, biochemical tests, serum electrolytes, coagulation screening, chest x-ray, electrocardiogram, heart Doppler ultrasound and so on. The localization of parathyroid glands was evaluated by using neck ultrasonography and parathyroid scintigraphy (termed Tc-99 m 2 methoxy-isobutyl-isonitrile, 99mTc-MIBI). Despite all this, identification of the exact location is still challenging. During the surgery, exploration for the hyperplasia parathyroid glands was performed with the patient under general anaesthesia, using a transverse collar incision. All patients underwent total parathyroidectomy without autotransplantation. All resected parathyroid glands were measured, weighed and verified histologically. The volume of each parathyroid gland (PTG) was estimated by using the formula: a * b * c *π/6 mm3 (where a, b, and c are the dimensions of gland in millimeters).

### Analysis of laboratory values

Blood routine tests were performed using an LH-750 Hematology Analyzer (Beckman Coulter, Inc., Fullerton, CA). Biochemical indices, such as serum albumin, calcium (Ca), phosphorus (P), and alkaline phosphatase (ALP), were measured using an Automatic Biochemical Analyzer (HITACHI 7080; Hitachi, Ltd, Tokyo, Japan). Serum intact-parathyroid hormone (iPTH) levels were measured using a UniCel DxI800 Access Immunoassay System (Beckman Coulter, Inc., Fullerton, CA).

### Statistical analyzes

Statistical analysis was done with SPSS 15 J for Windows (SPSS, Chicago, IL, USA) to observe any significant differences. All data is shown as the mean ± S.D., unless otherwise indicated. The relationship between the intact PTH levels and the weight, the volume, the maximum diameter of parathyroid glands were investigated by Spearman’s rank correlation coefficient. P-values <0.001 were considered significant.

## Results

### Clinical parameters of hemodialysis (HD) patients undergoing parathyroidectomy

**Fig. 1:**
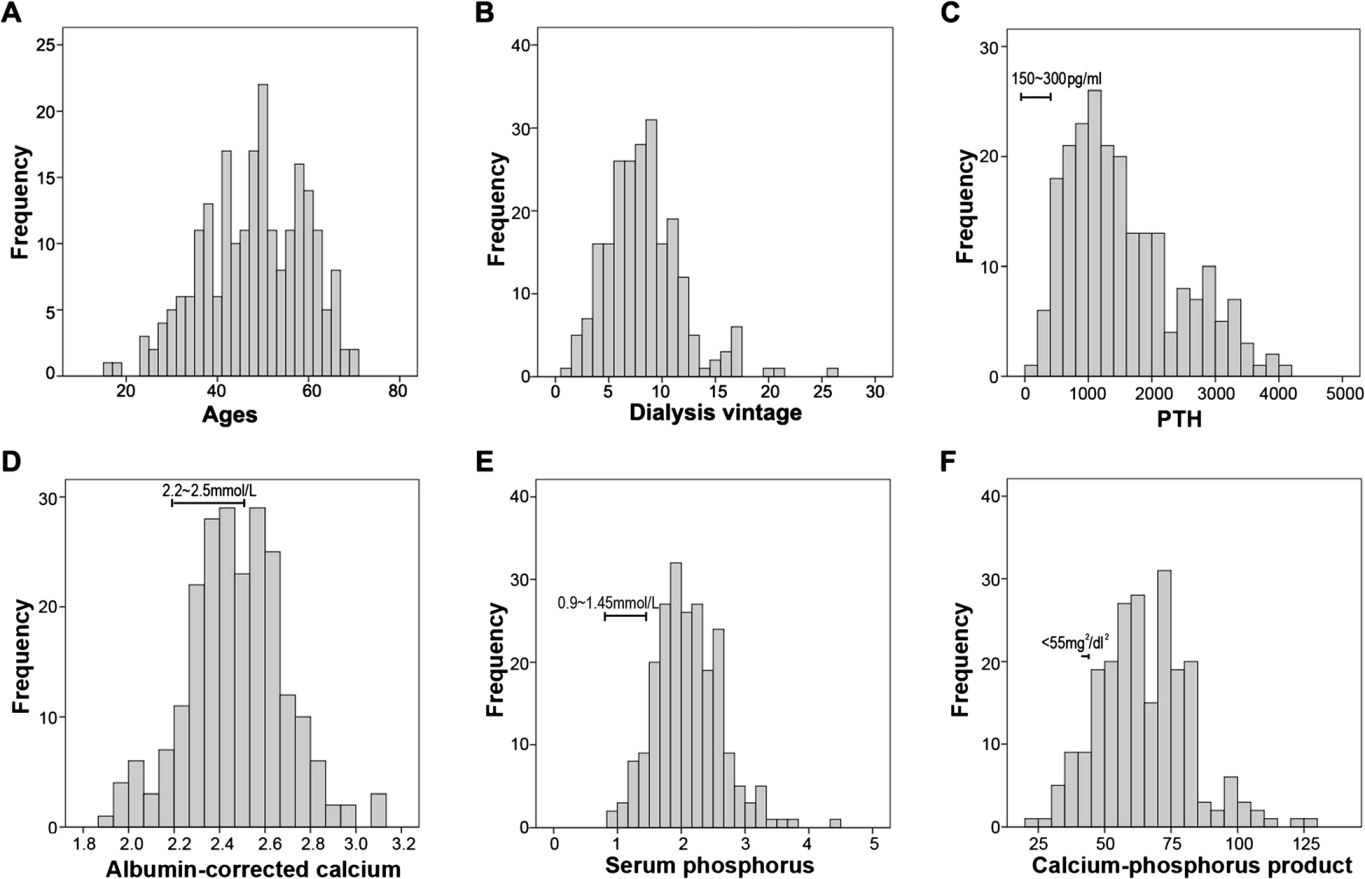
Histogram for the frequency distribution of ages, dialysis vintages, PTH levels, albumin-corrected calcium, serum phosphorus and calcium-phosphorus product in 223 patients with secondary hyperparathyroidism. a: age distribution patterns of patients; b: distribution of cases according to dialysis vintages; c: distribution of cases according to PTH levels; d: distribution of cases according to albumin-corrected calcium levels; e: distribution of cases according to serum phosphorus levels; f: distribution of cases according to calcium-phosphorus product

As shown in Table 1, the patients’ ages ranged from 16 to 70 years (average, 47.6 ± 11.11 years); They had undergone maintenance hemodialysis therapy for 1 ~ 26 years (average, 8.3 ± 3.64 years). 126 patients were men and 97 were women. The frequency distribution can be seen in Fig. 1.

**Table 1:** Patient characteristics

Characteristic	Patients (n = 223)	Normal range
Age, years	47.6 ± 11.11	-
Gender (male/female)	126/97	-
Dialysis vintage, years	8.3 ± 3.64	-
Kt/V	1.4 ± 0.19	1.2 ~ 1.8
Calcium,mmol/L	2.51 ± 0.23	2.15 ~ 2.55 mmol/L
Phosphorus, mmol/L	2.11 ± 0.52	0.81 ~ 1.45 mmol/L
albumin-corrected calcium, mmol/L	2.47 ± 0.22	-
Albumin, g/dl	41.8 ± 4.37	39.7 ~ 49.4 g/L
calcium-phosphorus product	64.9 ± 17.50	-
Alkaline phosphatase (U/l)	405.5 ± 423.97	40 ~ 129 U/L
Intact PTH, pg/ml (preoperative)	1556.9 ± 879.27	12 ~ 88 pg/ml
Intact PTH, pg/ml (postoperative 1st day)	16.0 ± 44.76	12 ~ 88 pg/ml

Data are expressed as mean ± S.D

Based on the criteria suggested by Gagne et al. [15], surgical procedure was considered successful in all patients whose serum levels of iPTH dropped from mean values of 1556.9 ± 879.27 pg/ml to 16.0 ± 44.76 pg/ml (ANOVA, *P* < 0.001). More than 90 % decline in 24 h-postoperative PTH reliably predicts operative success.

### Correlation of the weight with the size of parathyroid gland

**Fig. 2:**
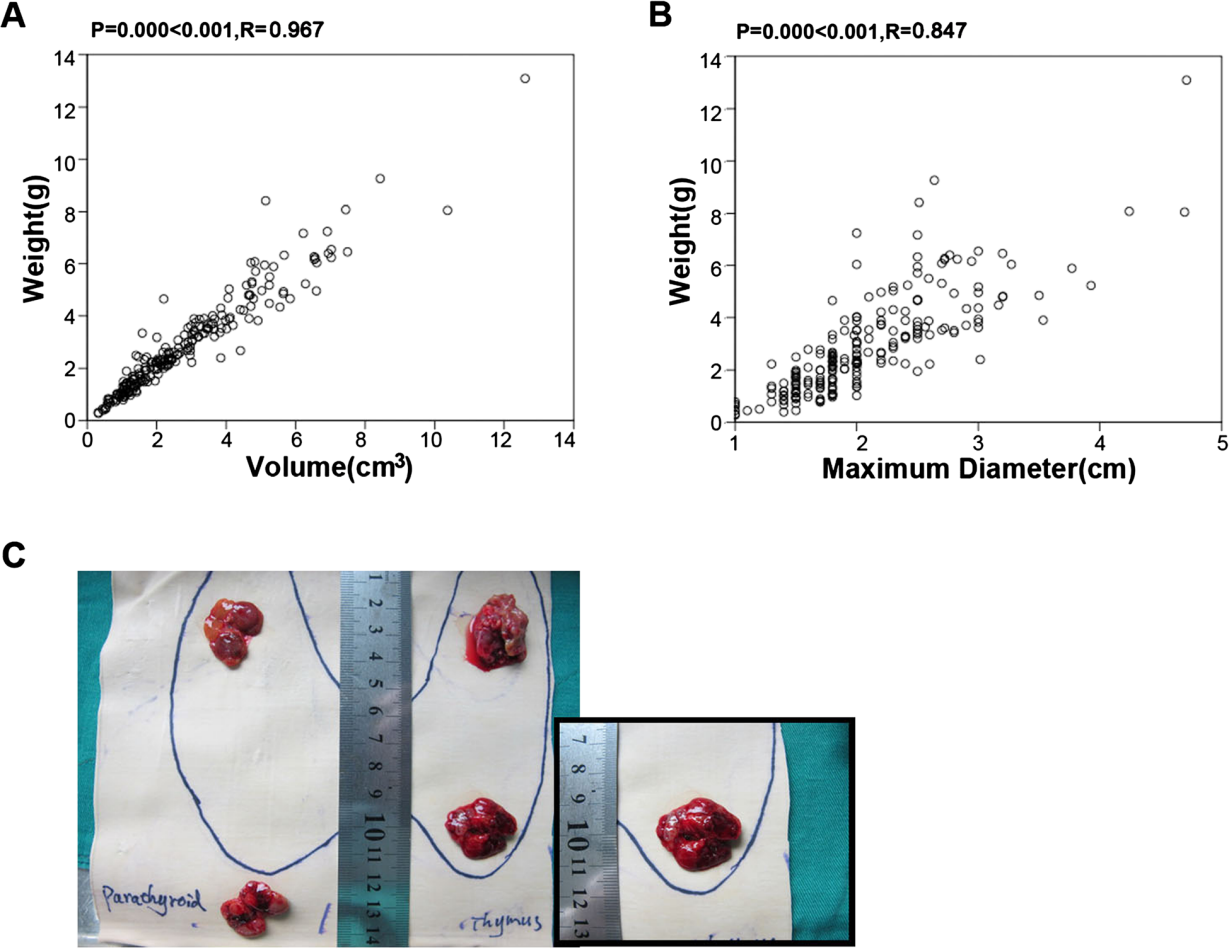
The relationship between the weight of parathyroid glands and their volume (n = 223). **a** and **b**: A positive correlation was found between the total weight of the glands and their sizes. **c**: The parathyroid glands identified and measured at the primary operation in patient for secondary hyperparathyroidism

In 223 patients, 874 glands were resected (Table 2). 214 patients had 4 parathyroid glands. Three glands were removed in 8 patients. As shown in Fig. 2c, the sizes of parathyroid gland were measured. By histopathology, all patients had the diagnosis of nodular parathyroid hyperplasia. After operation, pruritus, bone pain and muscle weakness disappeared.

**Table 2:** 874 parathyroid glands identified at the primary operation in 223 patients for secondary hyperparathyroidism

Location	No. of parathyroid glands	The size (cm)	The volume (cm^3^)	The weight (g)
Right superior parathyroid gland	214	1.4 ± 0.57 (0.5–3.5)	0.35 ± 0.69 (0.01–4.24)	0.40 ± 0.65 (0.01–3.84)
Right inferior parathyroid gland	221	1.6 ± 0.48 (0.6–2.8)	0.60 ± 0.68 (0.06–3.27)	0.63 ± 0.69 (0.03–3.7)
Left superior parathyroid gland	221	1.5 ± 0.50 (0.4–3)	0.38 ± 0.60 (0.02–4.71)	0.40 ± 0.62 (0.01–4.55)
Left inferior parathyroid gland	218	1.6 ± 0.51 (0.5–3)	0.59 ± 0.81 (0.03–4.70)	0.67 ± 0.73 (0.04–4.32)

Data are expressed as mean ± S.D

We calculated the total weight and volume of all resected glands for each patient. As shown in Fig. 2a and b, a positive correlation was identified between the mass and the weight of resected parathyroid glands (*P* < 0.001, *R* = 0.967 for the volume; *P* < 0.001, *R* = 0.847 for the maximum diameter;).

### Correlation of the preoperative intact-PTH and the reduction of intact-PTH with the size of parathyroid gland

**Fig. 3:**
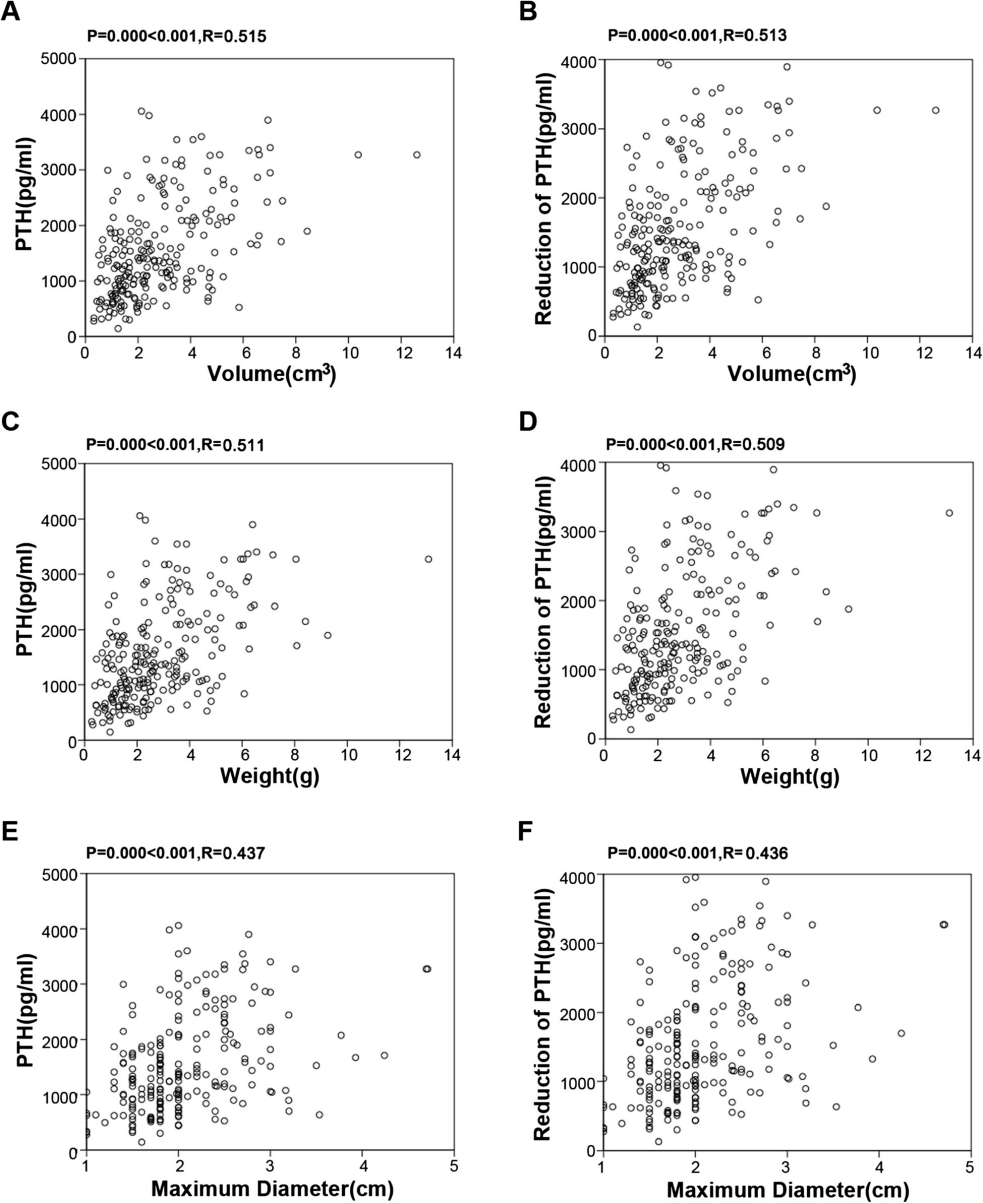
The relationship between the levels of intact PTH and the parathyroid gland mass (n = 223). **a**, **c** and **e**: The level of iPTH was correlated with the resected parathyroid gland mass. **b**, **d** and **f**: The reduction of iPTH was correlated with the resected parathyroid gland mass

Figures 3a, c and e show the correlation of serum intact PTH with the volume, the weight, and the maximum diameter of parathyroid gland (*P* < 0.001, *R* = 0.515; *P* < 0.001, *R* = 0.511; *P* < 0.001, *R* = 0.437; respectively). A positive correlation was also observed between the reduction of intact PTH after surgery and the volume, the weight, and the maximum diameter of parathyroid gland (*P* < 0.001, *R* = 0.513; *P* < 0.001, *R* = 0.509; *P* < 0.001, *R* = 0.436; respectively). Therefore, the increased parathyroid mass seems to secrete more PTH.

Then, we divided the patients into 3 subgroups: PTH < 500 pg/ml, 500 ~ 1000 pg/ml and PTH > 1000 pg/ml. In the subgroups of patients with PTH < 500 pg/ml and PTH < 1000 pg/ml, no significant correlation was found between PTH levels and parathyroid gland (Table 3). Whereas, in the subgroups of patients with PTH > 1000 pg/ml, serum intact PTH was correlated significantly in a positive manner with the parathyroid gland mass (*P* < 0.001, *R* = 0.338; *P* < 0.001, *R* = 0.396; *P* < 0.001, *R* = 0.404; respectively).

**Table 3:** Bivariate Pearson’s correlation analysis in three subgroups of hemodialysis patients with different serum PTH levels

PTH level (pg/ml)	PTG Volume (cm^3^)		PTG Weight (g)		PTG Maximum Diameter (cm)	
	P value	Spearman’s rho	P value	Spearman’s rho	P value	Spearman’s rho
<500	0.592	0.172	0.957	0.018	0.948	0.021
500 ~ 1000	0.297	0.140	0.186	0.178	0.248	0.156
>1000	0.000*	0.338	0.000*	0.396	0.000*	0.404

*P < 0.001, statistically significant

## Discussion

Chronic kidney disease–mineral and bone disorder (CKD-MBD) is a growing health care concern associated with secondary hyperparathyroidism, mineral abnormalities, and increased risk of cardiovascular disease [16, 17]. As one of the most common abnormalities of CKD-MBD, secondary hyperparathyroidism leads to nodular transformation of the parathyroids in up to 75 % of cases [18, 19]. Can the increased glandular mass be reduced? Such a reduction would call for massive apoptosis to take place in the parathyroids. However, there are no known stimuli for apoptosis in the parathyroid cells [9]. Thus, poor efficacy of pharmaceutical drug in progressive secondary hyperparathyroidism (SHPT) argues in favour of parathyroidectomy [20–22]. The problem is particularly severe in China. That’s because vitamin D analogs and calcimimetics were unavailable in mainland China during the study period. The most common drugs currently used to treat secondary hyperparathyroidism in China were calcium-based phosphorus binders and non-selective VDR activator medications which may cause recurrent hypercalcemia and hyperphosphataemia.

Since the sensitivity of imaging procedures has been reported to be lower in secondary hyperparathyroidism than in primary hyperparathyroidism [23], even with the use of combined neck ultrasonography and parathyroid scintigraphy, bilateral surgical exploration with identification of all glands is required. What’s more, since the relatively small size of missed adenomatous glands could always lead to a high rate of re-operation in whom major anaesthesia risks are frequent, the surgeons need to get a general preoperative assessment not only about the localization but also the influence of parathyroid mass on PTH secretion.

The relationship with the parathyroid gland and the parathyroid hormone level was still uncertain until now. Inaba et al. reported that serum bio-PTH but not intact PTH correlated significantly in a positive manner with the maximal diameter of the parathyroid gland [23]. Takatoshi et al. also demonstrated that the secretion of PTH solely depends on the size of the parathyroid glands [24]. However, some evidence hints that the parathyroid mass is to a large extent of less importance for PTH secretion than the ‘quality’ of parathyroid mass. The ‘content’ of the different noduli in severe parathyroid hyperplasia is determining the PTH secretion [9]. Since the mass of the parathyroid gland was usually determined by ultrasonography in those studies, the ultrasonography tests are usually heavily operator-dependent and show a wide variability. In our study, we evaluated the mass by directly measuring the three dimensions of the resected parathyroid glands in operation. We found that nodular transformation of the parathyroids was up to 90 % in our cases. Furthermore, a significantly positive correlation between the resected parathyroid gland and the intact serum PTH was found. This data suggested that the larger parathyroid gland might secrete more intact PTH, confirming that the size of a gland could be used as an indication for surgical treatment. However, in the subgroup patients with iPTH < 1000 pg/ml, no significant correlation was found. The difference might lie in the previous treatment with phosphate binders, vitamin D analogs or calcimimetics. However, large evidence confirmed that PTH levels might return to pre-treatment values when the treatment is stopped [24–26]. What’s more, with the condition progress, medicine resistance could be a result of the reducing expression of calcium-sensing receptors (CaSR) and vitamin D receptors (VDR). Thus, we cannot help wondering should we still use repeated non-selective VDR activator in poorly controlled secondary hyperparathyroidism. Our results suggested that PTH levels might be influenced by the pharmaceutical drug under manageable condition, whereas the size of parathyroid gland is to a large extent of more importance on the regulation of PTH secretion. Therefore, the large size of parathyroid gland might be used as a much more appropriate guide that indicates the requirement of surgery treatment even when the parathyroid hormone was less than 1000 pg/ml.

## Conclusion

In summary, nodular transformation of the parathyroids was common in the patients with severe secondary hyperparathyroidism. Generally, larger parathyroid gland secretes more PTH and high level of serum PTH usually indicated that surgical removal might be required. However, since PTH levels could easily be influenced by the pharmaceutical drug, the large size of parathyroid gland might be used as a much more appropriate guide that indicates the requirement of surgery treatment.

## Abbreviations

PTH: Parathyroid hormone
CaSR: Calcium-sensing receptors
VDR: Vitamin D receptors
PTX: Parathyroidectomy
EDTA: Ethylene diamine tetraacetic acid
99mTc-MIBI: Tc-99 m methoxy-isobutyl-isonitrile
PTG: Parathyroid gland
Ca: Calcium
P: Phosphorus
ALP: Alkaline phosphatase
iPTH: Intact-parathyroid hormone
CKD-MBD: Chronic kidney disease–mineral and bone disorder
SHPT: Secondary hyperparathyroidism
